# Supplementary material for: The Polycomb protein Ezl1 mediates H3K9 and H3K27 methylation to repress transposable elements in Paramecium
Source: Nat Commun. 2019 Jun 20;10:2710. doi: 10.1038/s41467-019-10648-5 (PMC6586856; doi:10.1038/s41467-019-10648-5)
Supplement: Supplementary file 1 — Supplementary Information [file 41467_2019_10648_MOESM1_ESM.pdf]

Supplementary Information

The Polycomb protein Ezl1 mediates H3K9 and H3K27 methylation to repress transposable elements in *Paramecium*

Andrea Frapporti, Caridad Miró Pina, Olivier Arnaiz, Daniel Holoch, Takayuki Kawaguchi, Adeline Humbert, Evangelia Eleftheriou, Bérangère Lombard, Damarys Loew, Linda Sperling, Karine Guitot, Raphaël Margueron and Sandra Duharcourt

a

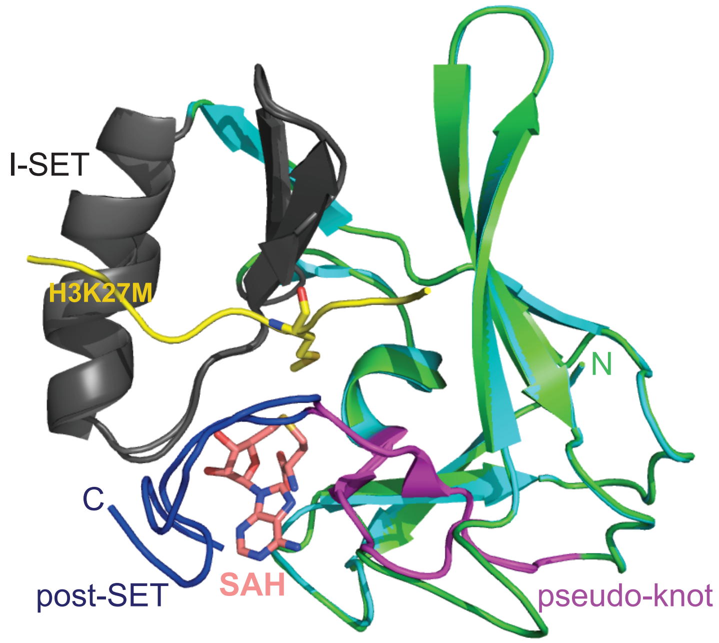

b

```
                             SET                                     I-SET
Ezl1 442 MQIRKSLVLG KSLICNGLGL FAAQNFKVCD FVGEYTGNYI LLDDESMAIE QCDWITNNH- 501
Ezh2 608 RGSKKHLLLA PSDVA-GWGI FIKDPVQKNE FISEYCGE-I ISQDEADRRG KVYDKYMCS- 664
Dim5 145 RGRTVPLQIF RTKDR-GWGV KCPVNIKRGQ FVDRYLGE-I ITSEEADRRR AESTIARRKD 202
            I-SET                        *                  SET
Ezl1 502 -YLFEVDDK- ---------- -----WQVDG TYYSNCLRYI NHATKKSDLA NCQAQILFSE 544
Ezh2 665 -FLFNLNND- ---------- -----FVVDA TRKGNKIRFA NHSVN----P NCYAKVMMVN 703
Dim5 203 VYLFALDKFS DPDSLDPLLA GQPLE--VDG EYMSGPTRFI NHSCD----P NMAIFARVGD 256
                       SET                                  post-SET
Ezl1 545 G----RWRIA MFTTKNISIG EELFFDYGD- -KFLTKWLTD FNKLCDDYYK K----- 589
Ezh2 704 G----DHRIG IFAKRAIQTG EELFFDY--- -RYSQADAL- -------KYVG IE---- 740
Dim5 257 HADKHIHDLA LFAIKDIPKG TELTFDYVNG LTGLESDAHD PSKISEMTKC LCGTAK 312
```

Supplementary Figure 1. Comparative sequence and homology modeling of the catalytic SET domain of *Paramecium tetraurelia* Ezl1 and human Ezh2.
a Structural alignment of human Ezh2 SET domain (cyan) (PDB ID: 5HYN) bound to the peptide inhibitor H3K27M (yellow) and to SAH cofactor (pink) with modeled *Paramecium tetraurelia* Ezl1 (green). The pseudoknot-like structure, the I-SET domain and the post-SET domain (unaligned) are displayed. **b** Sequence alignement of *P. tetraurelia* Ezl1 (PTET.51.1.G1740049) with human Ezh2 (Q15910) and *Neurospora crassa* Dim-5 (AF419248). Substrate and cofactor binding residues are highlighted in yellow and green, respectively. Important residues for catalysis are highligted in red. Identical residues are colored in grey and positively substituted residues in light grey. Position A677 in Ezh2, which corresponds to a glycine in both Ezl1 and Dim-5, is indicated by an asterisk.

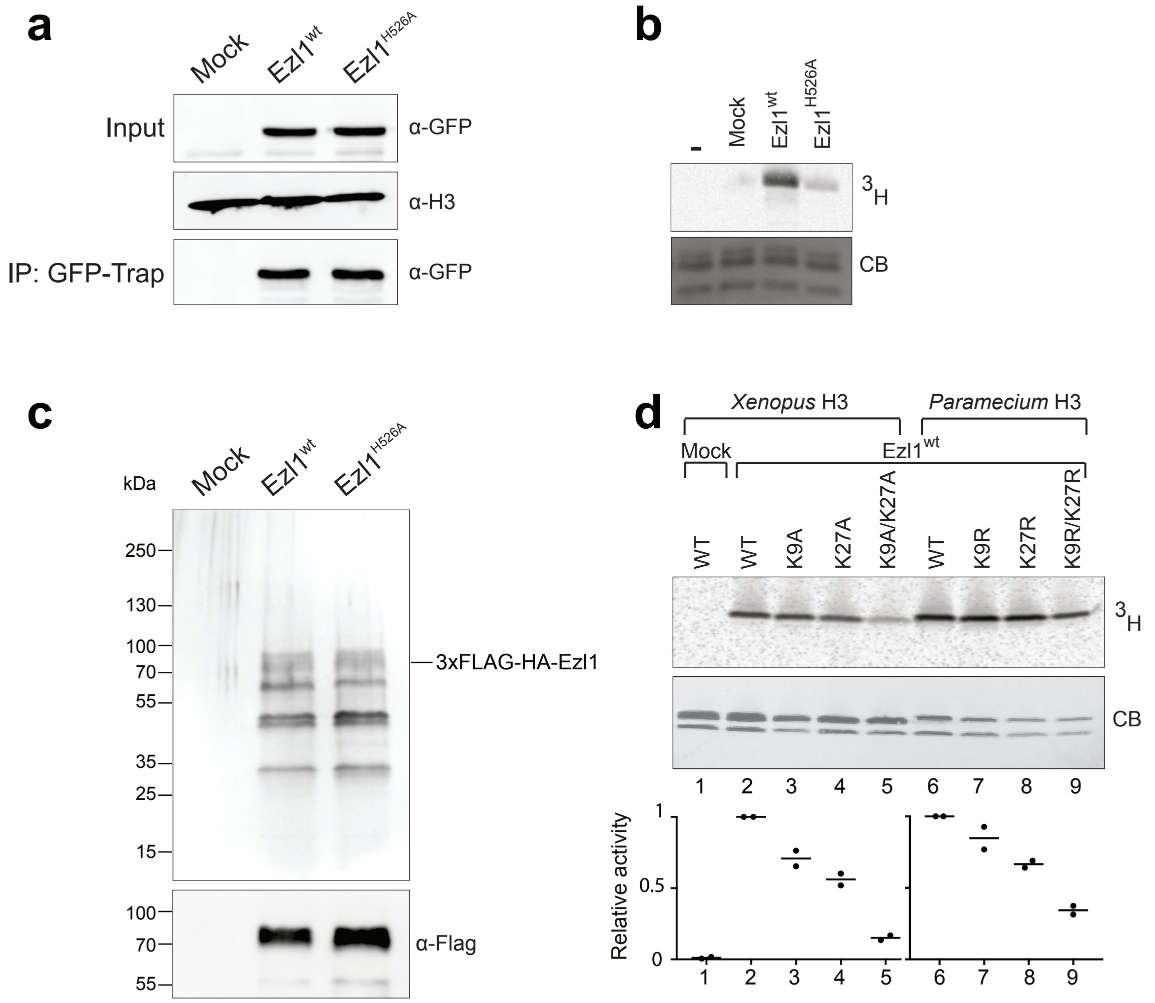

e

	4 9 23 27
Xenopus H3	ARTKQTARKSTGG-KAPRKQLATKAARKSAP
Paramecium H3	ARTKQTARKSTAGNKKPTKHLATKAARKTAP

Supplementary Figure 2. Immunoprecipitations of tagged-Ezl1 fusion proteins from nuclear extracts used for *in vitro* histone methyltransferase assays.

a (Top panels) Western-blot analysis of *Paramecium* nuclear extracts prepared 12 hours after the onset of sexual events in non-injected (mock) cells, cells transformed with *GFP-EZL1^{wt}* or *GFP-EZL1^{H526A}* RNAi-resistant transgenes upon *EZL1* RNAi, with anti-GFP and anti-H3 antibodies. Anti-H3 antibody shows comparable amounts of nuclear extracts that were used as inputs for immunoprecipitation with GFP-Trap_M beads. (Bottom panel) Western-blot analysis of GFP-Trap immunoprecipitates used for histone methyltransferase assays (Fig. 2a-c and panel b), with anti-GFP antibodies. **b** Immunoprecipitates from panel a were used for *in vitro* histone methyltransferase reactions with wild type *Xenopus* recombinant histone octamers as substrates and S-adenosyl-[methyl ^{3}H]-methionine as methyl donor. The same reactions with histone octamers only were performed as a negative control. The reaction products were separated by SDS-PAGE and transferred onto a PVDF membrane. Coomassie stain (CB, bottom panel) shows histones and the autoradiograph (^{3}H, top panel) indicates H3 histone methyltransferase activity. **c** Double affinity pull-down of 3xFLAG-HA-Ezl1^{wt} or 3xFLAG-HA-Ezl1^{H526A} proteins from nuclear extracts prepared 12 hours after the onset of sexual events in non-injected (mock) cells, or transformed *Paramecium* cells, depleted for the endogenous Ezl1 protein in the case of 3xFLAG-HA-Ezl1^{H526A}. Top panel: Silver-stained gel of pulled-down proteins used for *in vitro* histone methyltransferase assays (Fig. 2d-e and panel d). Bottom panel: Western-blot analysis with anti-FLAG antibodies. **d** Immunoprecipitates from panel c were used for *in vitro* histone methyltransferase assays with recombinant histone octamers assembled with *Xenopus* H3 or tetramers assembled with *Paramecium* H3 as substrates. The graphs show relative quantification of histone methyltransferase signals analyzed by scintillation counting after SDS-PAGE. Circles indicate the individual data points. Horizontal bars represent the mean of two biological replicates. More residual activity appears detectable with the *Paramecium* double mutant H3 (lane 9) than with the *Xenopus* double mutant H3 (lane 5). This difference may be due to the fact that the mutants are not exactly the same (K9A/K27A for *Xenopus* and K9R/K27R for *Paramecium*) or to differences in the N-terminal amino acid sequence between the two proteins (see panel e), or both. **e** Sequence alignment of the N-terminal *Xenopus* and *Paramecium* H3 (H3P1) proteins. Note the insertion of one (N) residue at position 14 in the *Paramecium H3* sequence, resulting in one amino acid gap between positions in the *Xenopus* and *Paramecium* sequences (Table 1 and Supplementary Data 1). For simplicity, K24 and K28 in the *Paramecium* H3 sequence are referred to as K23 and K27, respectively. According to quantitative label free mass spectrometry analysis (Fig. 2e, Table 1 and Supplementary Data 1), K9 and K27 (highlighted in yellow) are methylated in an Ezl1-dependent manner. Source data are provided as a Source Data file.

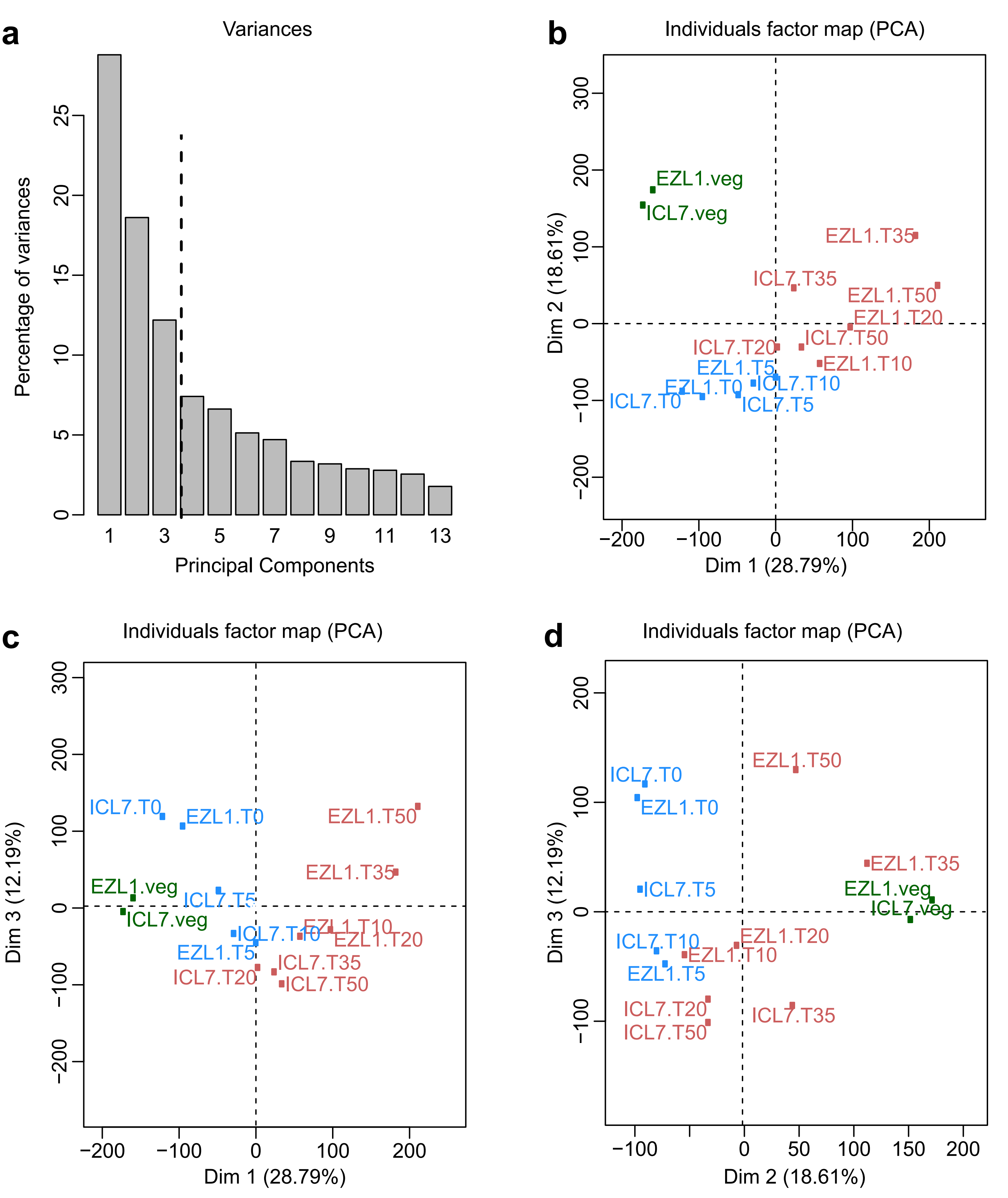

Supplementary Figure 3. Principle Component Analysis (PCA) clustering of RNA-seq samples.
Normalized fragment counts for all elements and all samples were analyzed by PCA. This technique finds coordinate systems that explain the variance in the data. **a** Barplot showing the variability accounted for by the PCA components. **b** Biplot representing the RNA-seq samples on the coordinate system defined by PCA components 1 and 2, representing 47% of global variance, that separates the samples into 3 groups, corresponding to vegetative stage (ICL7 and EZL1 vegetative samples, in green), Early developmental stage (ICL7: T0, T5, T10; EZL1: T0, T5 samples, in blue) and Late developmental stage (ICL7: T20, T35, T50 and EZL1: T10, T20, T35, T50). This is the sample grouping used for all analyses. **c** Biplot representing the samples on the coordinate system defined by PCA components 1 and 3. **d** Biplot representing the samples on the coordinate system defined by PCA components 2 and 3.

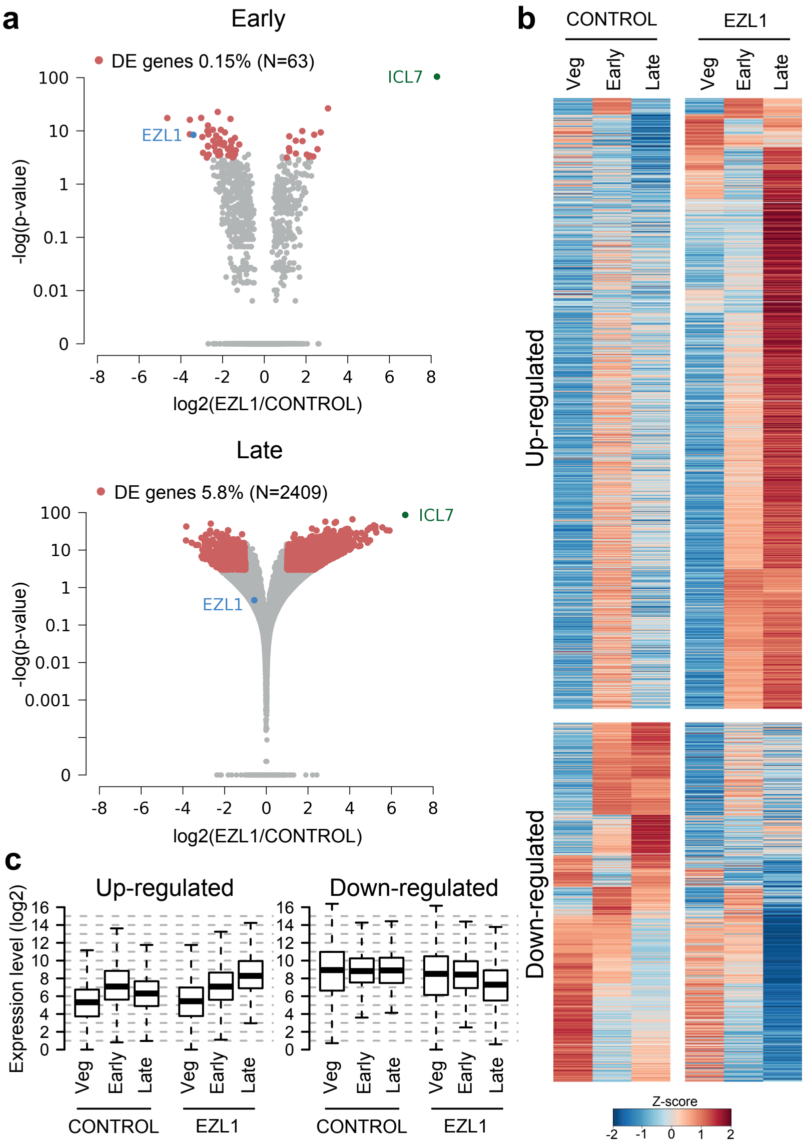

Supplementary Figure 4. Gene expression upon *EZL1* RNAi.
a Volcano plot representation of up- and down-regulated genes between *EZL1* and *ICL7* (control) RNAi for Early (top) and Late (bottom) developmental stages as measured by RNA-seq. Red dots indicate significantly misregulated genes between the two conditions (fold change >2 and p-value <0.05): 63 genes at Early stage (0.15% of all genes) and 2,409 genes at Late stage (5.8% of all genes). At the Early stage, 0.04% of all genes are up-regulated and 0.11% are down-regulated. At the Late stage, 3.7% of all genes are up-regulated and 2.1% down-regulated. The *ICL7* gene and the *EZL1* gene are shown in green and blue, respectively. They attest to knockdown efficiency: *EZL1* is much less expressed in the *EZL1* RNAi than in the *ICL7* control RNAi at the Early stage, when *EZL1* expression peaks. Conversely, the constitutively expressed gene *ICL7* is much less expressed in the control (*ICL7*) RNAi than in *EZL1* RNAi at both the Early and Late stages. **b** Heatmap ordered by hierarchical clustering of EZL1 and ICL7 developmental stages (see Methods) of up- and down-regulated genes between *EZL1* and *ICL7* (control) RNAi as measured by RNA-seq. Each row represents a gene and each column a developmental stage (Veg, vegetative; Early; Late) for *EZL1* (left) and *ICL7* (right) RNAi. The Z-score color, from dark blue to dark red, represents the number of standard deviations from the mean expression across all 6 conditions. **c** Boxplot representation of normalized expression levels for up-regulated and down-regulated genes presented in the panel b.

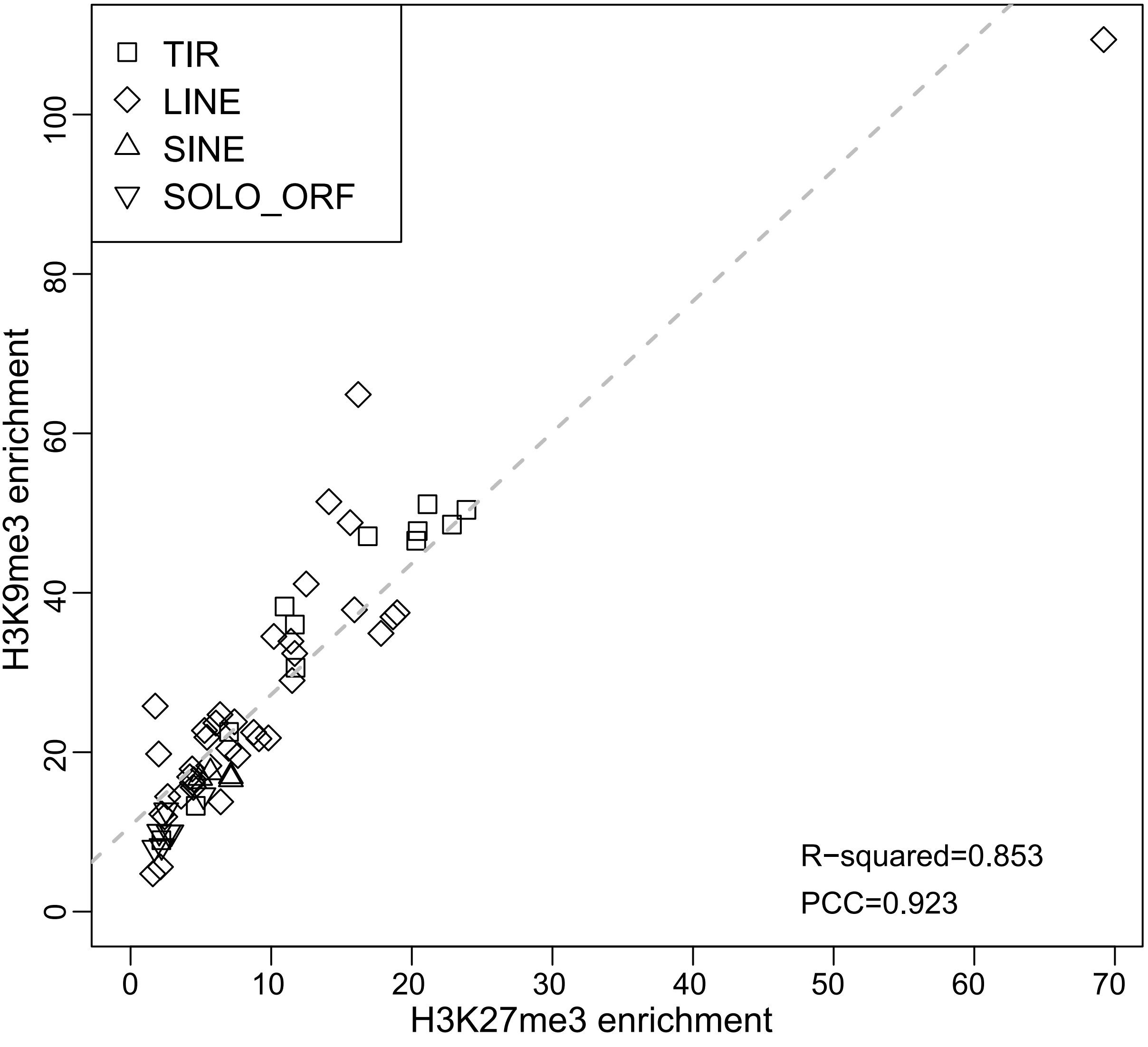

Supplementary Figure 5. Correlation of enrichment for H3K27me3 and H3K9me3 on TE families. Pearson correlation coeficient (PCC) and R^2 between H3K9me3 and H3K27me3 enrichment relative to input for the 61 TE families presented in Fig. 5c are indicated.

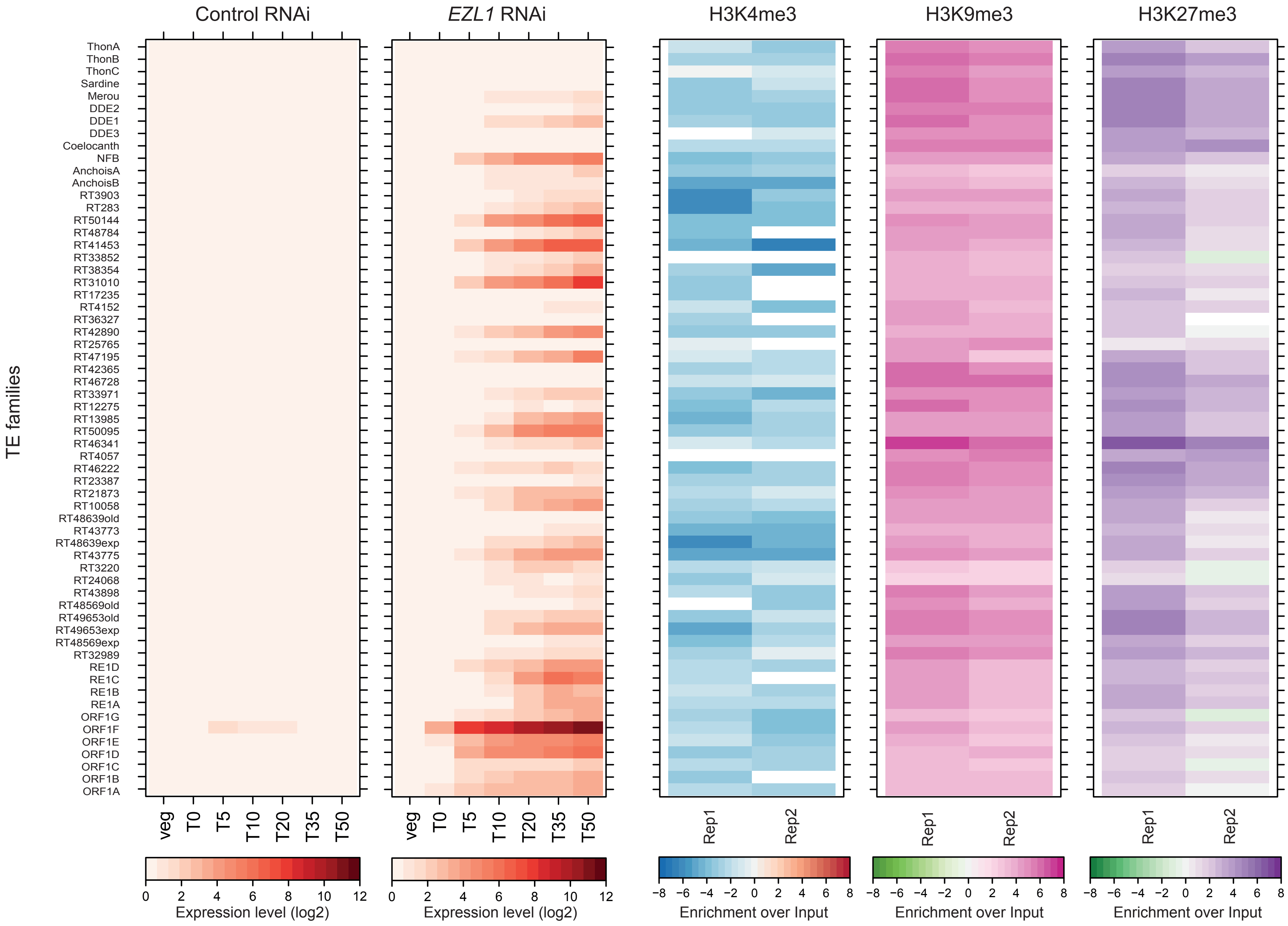

Supplementary Figure 6. Heatmap of RNA levels and histone marks enrichment for 61 TE families.
(Left panels) Heatmaps of RNA expression levels for 61 TE families are shown at different time points during development upon control (ICL7) and EZL1 RNAi. Each row represents a TE family and each column a time point during an autogamy time course (see Supplementary Fig. 3). (Right panels) Heatmaps of H3K4me3, H3K9me3 and H3K27me3 enrichment over input for the 61 TE families, for each biological replicate presented in Fig. 5c.

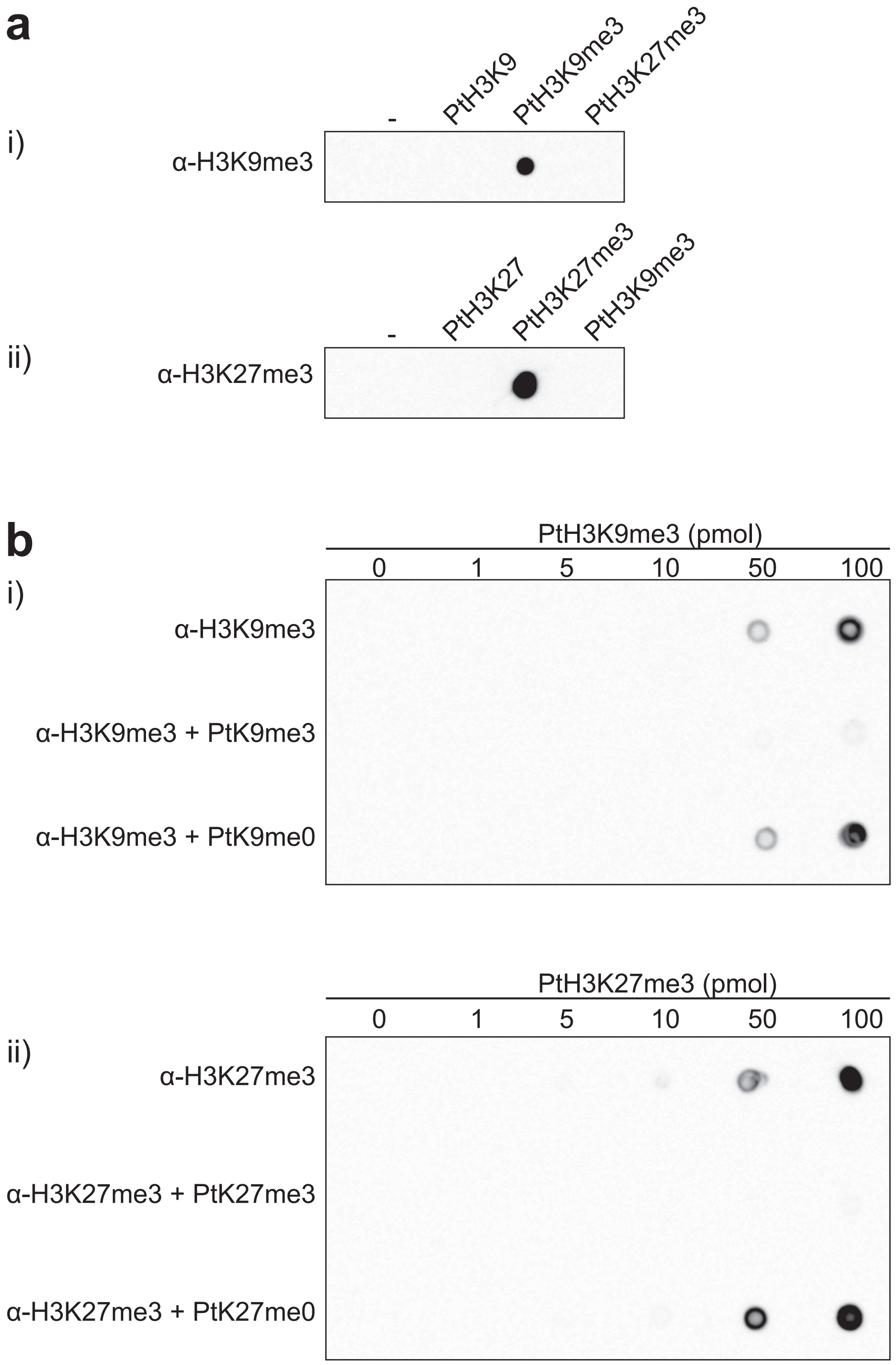

Supplementary Figure 7. Specificity of H3K9me3 and H3K27me3 antibodies.
a Dot blot assay using *Paramecium tetraurelia* H3 peptides. 100 ng of each of the indicated peptides was spotted on the membrane. No peptide was spotted for the negative control (-). i) Hybridization with the H3K9me3 polyclonal antibodies showed specific reactivity with tri-methyl K9 (PtH3K9me3: CKQTARK(me3)STAGN) but not against the unmodified peptide nor the tri-methyl K27 (PtH3K27me3: TKAARK(Me3)TAP). ii) Hybridization with the H3K27me3 polyclonal antibodies showed specific reactivity with tri-methyl K27 but not against the unmodified peptide nor the tri-methyl K9. **b** Competition assay. 0 to 100 pmol of *Paramecium* H3K9me3 (i) or H3K27me3 (ii) peptides were spotted and probed with the H3K9me3 (i) or H3K27me3 (ii) antibodies alone or in presence of a 100-fold molar excess of the indicated peptides. Pre-absorption with the PtH3K9me3 (i) or PtH3K27me3 (ii) peptides dramatically reduces the signal, while pre-absorption with the un-methylated peptide has no effect. Source data are provided as a Source Data file.

Stage	Label	ENA Accession	Number of reads	Number of mapped reads on MAC	%	Number of mapped reads on MIC	%
**VEG**	EZL1.veg	ERX2078722	49394256	46878066	95	113835	0
**EARLY**	EZL1.T0	ERX2078723	108981514	102588411	94	467396	0
	EZL1.T5	ERX2078724	141404920	118242226	84	749587	1
	EZL1.T10	ERX2078725	57380782	55043791	96	592750	1
**LATE**	EZL1.T20	ERX2078726	59605368	55678537	93	2041696	3
	EZL1.T35	ERX2078727	24060916	19693556	82	3292949	14
	EZL1.T50	ERX2078728	64896124	52192723	80	9486842	15
**VEG**	ICL7.veg	ERX2078729	92371700	87012622	94	239508	0
**EARLY**	ICL7.T0	ERX2078730	85117070	80131612	94	341632	0
	ICL7.T5	ERX2078731	89067894	84074611	94	388490	0
**LATE**	ICL7.T10	ERX2078732	83730830	78744105	94	376792	0
	ICL7.T20	ERX2078733	89608604	83504251	93	326938	0
	ICL7.T35	ERX2078734	47982586	45581393	95	152053	0
	ICL7.T50	ERX2078735	79970902	73891476	92	301197	0

Supplementary Table 1. Description of RNA-seq data deposited in the European Nucleotide Archive.
The columns provide the stage, the sample label, the ENA accession, the total number of reads, the number of reads that were mapped to the MAC reference genome and the number of reads that did not map to the MAC reference that did map to the MIC assembly. The percentage of mapped reads is with respect to the total number of reads in each sample.

	All annotated genes		Differentially Expressed genes				Up-regulated genes				Down-regulated genes			
	Number	Perc.	Number	Perc.	Chi2	Chi2 Pvalue	Number	Perc.	Chi2	Chi2 Pvalue	Number	Perc.	Chi2	Chi2 Pvalue
**Number of coding genes**	40460		2375				1505				870			
**Autogamy coding genes**	17190	42.5%	1519	64.0%	420	1.98e-93	926	61.5%	214	1.47e-48	593	68.2%	229	9.75e-52
Intermediate peak	2037	5.0%	451	19.0%	799	1.14e-175	438	29.1%	1515	<1e-324	13	1.5%	23	1.95e-06
Late induction	3741	9.2%	359	15.1%	89	3.4e-21	276	18.3%	139	5.4e-32	83	9.5%	0,09	0.767
Early peak	1974	4.9%	136	5.7%	3	0.0636	119	7.9%	28	1.17e-07	17	2.0%	16	6.71e-05
Late peak	468	1.2%	67	2.8%	50	1.26e-12	24	1.6%	2	0.121	43	4.9%	100	1.55e-23
Early repression	4536	11.2%	259	10.9%	0.2	0.646	41	2.7%	108	3.37e-25	218	25.1%	160	9.21e-37
Late repression	4434	11.0%	247	10.4%	0.7	0.396	28	1.9%	126	2.5e-29	219	25.2%	172	2.42e-39

Supplementary Table 2. DE genes upon *EZL1* RNAi are enriched in autogamy genes.
Six gene expression profiles during autogamy (Intermediate peak, Late induction, Early peak, Late peak, Early repression and Late repression) were defined in Arnaiz et al. 2017, involving 42% (N=17190) of all annotated coding genes (N=40460). The table presents a comparison of percentages of three datasets of DE genes upon *EZL1* knockdown (DE expressed genes, Up-regulated DE genes, Down-regulated DE genes) with the percentages for all annotated coding genes in each expression profile group. The significance is tested using a Chi-square test. The Chi2 and P-value statistics are shown for each comparison. The DE genes upon *EZL1* knockdown are enriched significantly in autogamy genes and more specifically in genes from the Intermediate peak cluster.

	EZL1 RNAi	control RNAi (*ICL7*)	Fold-change
TE	311.4	1.1	282.52
genes	1715.9	355.5	4.83

Supplementary Table 3. Comparison of aggregate fold-change in differential expression (DE) for TE and for genes.
The total normalized RNA-Seq read counts per Kb for all Late stage DE TE and for all Late stage up-regulated DE genes were calculated (first 2 columns of table). The ratio of *EZL1* RNAi/control gives the average fold-change in the 3rd column, showing that TE de-repression is much more dramatic than gene DE.

Name	Sequence (5’ to 3’)	Locus<br>(ID)
**Actin_qPCR_for**	TGAAGCTCCAATGAATCCAA	Actin 1-1<br>(PTET.51.1.G0130204)
**Actin_qPCR-rev**	TCCTGAAGCATAGAGTGAGA	Actin 1-1<br>(PTET.51.1.G0130204)
**GAPDH_qPCR_F2**	ATTTTGGTATTGTTGAGGGT	GAPDH<br>(PTET.51.1.G0380195)
**GAPDH_qPCR_R2**	CTCCAGTCTTTTCCACCTTT	GAPDH<br>(PTET.51.1.G0380195)
**Oligo #751**	CTTAGTGGGGTAGAATGAGCA	EE PPase<br>(PTET.51.1.G1020193)
**Oligo #752**	GACTTCTGCTTTCTTTTCTGCA	EE PPase<br>(PTET.51.1.G1020193)
**Oligo #753**	GGAGAGGGAAAGATAAGAGT	ST PPase<br>(PTET.51.1.G1240023)
**Oligo #754**	CCACTCCTTGAATTTGAGGA	ST PPase<br>(PTET.51.1.G1240023)
**Oligo #759**	GAAGTAGGTATTATCGTGCC	ST Kinase<br>(PTET.51.1.G1270115)
**Oligo #760**	ACCATGTAAACAATTCAAGCA	ST Kinase<br>(PTET.51.1.G1270115)
**Oligo #769**	AGAGAGAGACTTCGTGATGA	Helicase<br>(PTET.51.1.G1070046)
**Oligo #770**	CAACTTGGGCATGTCAAAAT	Helicase<br>(PTET.51.1.G1070046)
**Anchois.173_F2**	TTCCAAGCTGATTCGATTATTAAA	Anchois B<br>(IESPGM.PTET51.1.173.70900)
**Anchois.173_R2**	ACTTCTTGTTTCATTTGTTAGACT	Anchois B<br>(IESPGM.PTET51.1.173.70900)
**IESA1835_F2**	GTGGATGGACTGGAACTTAA	IES 51A1835<br>(IESPGM.PTET51.1.106.284157)
**IESA1835_R2**	ACAATCCATCTATAAATGAGTT	IES 51A1835<br>(IESPGM.PTET51.1.106.284157)
**Oligo #727**	ACAAACGAACGAACAGATTG	RT50144<br>(ms5583_NODE_2217_length_6579<br>_cov_30.856514_RT50144_Group5_non-<br>LTR:ClassI:LINE)
**Oligo #728**	CTGAGATGGCATAACTCCTT	RT50144<br>(ms5583_NODE_2217_length_6579<br>_cov_30.856514_RT50144_Group5_non-<br>LTR:ClassI:LINE)
**Oligo #767**	TTATTATGAGGGTTGGCGTC	RT48784-1<br>(ms2529_NODE_45329_length_35351<br>_cov_22.766909_RT48784_Group5_non-<br>LTR:ClassI:LINE)
**Oligo #768**	TAACTACACGACACCAGATC	RT48784-1<br>(ms2529_NODE_45329_length_35351<br>_cov_22.766909_RT48784_Group5_non-<br>LTR:ClassI:LINE)
**Oligo #739**	GTAGCTTTAATGAACGCAGG	RT48784-2<br>(ms4165_NODE_5853_length_16457<br>_cov_20.256548_RT48784_Group5_non-<br>LTR:ClassI:LINE)
**Oligo #740**	TATAGAACCGCCACAATCAG	RT48784-2<br>(ms4165_NODE_5853_length_16457<br>_cov_20.256548_RT48784_Group5_non-<br>LTR:ClassI:LINE)
**Oligo #551**	ACAAGATTGACCAGGACTTATT	RT31010<br>(ms4410_NODE_3768_length_13900_cov<br>_21.582806_RT31010_Group4_non-<br>LTR:ClassI:LINE)
**Oligo #552**	ATATCATTCACTCCTGCAATCT	RT31010<br>(ms4410_NODE_3768_length_13900<br>_cov_21.582806_RT31010_Group4_non-<br>LTR:ClassI:LINE)

**Oligo #559**	TTAATTGAAGGCGAAGAAAGAC	RT42890 (ms1831_NODE_10132_length_49470 _cov_21.140064_RT42890_Group4_non-LTR:ClassI:LINE)
**Oligo #560**	ACCATTCAATATCTTCTCCCTC	RT42890 (ms1831_NODE_10132_length_49470 _cov_21.140064_RT42890_Group4_non-LTR:ClassI:LINE)
**Oligo #569**	TCGAAGTAGATTATGGCTGAAA	RT25765 (ms1973_NODE_322_length_47186 _cov_49.739605_RT25765_Group4_non-LTR:ClassI:LINE)
**Oligo #570**	CTTTCATCCTCCTCACTTAACT	RT25765 (ms1973_NODE_322_length_47186 _cov_49.739605_RT25765_Group4_non-LTR:ClassI:LINE)
**Oligo #563**	CAATTCCACCCAATATCAAACA	RT12275 (ms1017_NODE_8426_length_83307 _cov_43.323395_RT12275_Group3_non-LTR:ClassI:LINE)
**Oligo #564**	ATTAAAGGATGGTCAGAAAGCT	RT12275 (ms1017_NODE_8426_length_83307 _cov_43.323395_RT12275_Group3_non-LTR:ClassI:LINE)
**Oligo #735**	CCCAAGCAAAATCTGAAACA	RT43773 (ms1391_NODE_2993_length_64210 _cov_44.286140_RT43773_Group2_non-LTR:ClassI:LINE)
**Oligo #736**	CATTTTGGTGAGCACAGTTT	RT43773 (ms1391_NODE_2993_length_64210 _cov_44.286140_RT43773_Group2_non-LTR:ClassI:LINE)
**Oligo #761**	GACTATGCTGACGATCTTGT	RT48639-1 (ms6074_NODE_8863_length_4403 _cov_18.113333_RT48639old_Group2_non-LTR:ClassI:LINE)
**Oligo #762**	TTCTGATTGCCATAACACCA	RT48639-1 (ms6074_NODE_8863_length_4403 _cov_18.113333_RT48639old_Group2_non-LTR:ClassI:LINE)
**Oligo #723**	ATCATCTTTCCCTCACATCG	RT48639-2 (ms4963_NODE_3562_length_10237 _cov_31.527792_RT48639exp_Group2_non-LTR:ClassI:LINE)
**Oligo #724**	AGATTTCACGCTTCAGTTCT	RT48639-2 (ms4963_NODE_3562_length_10237 _cov_31.527792_RT48639exp_Group2_non-LTR:ClassI:LINE)
**Oligo #731**	TCCTCCATAAAACCAAACACT	RT32989 (ms2470_NODE_50533_length_36815 _cov_24.476028_RT32989_Group1_non-LTR:ClassI:LINE)
**Oligo #732**	ATTCTTCCGCCTTTGAAATG	RT32989 (ms2470_NODE_50533_length_36815 _cov_24.476028_RT32989_Group1_non-LTR:ClassI:LINE)
**Oligo #577**	CCATGTTTTCTCTATTTGGTCTAA	PTET.51.1.G1740048
**Oligo #578**	GTAGACAACACAGAAGTAATTTGA	PTET.51.1.G1740048
**Oligo #571**	CTTTTCACTGGTGTTGTTCC	GFP
**Oligo #572**	TCCATAAGTTGCATCACCTT	GFP

Supplementary Table 4. List of qPCR primers used in this study.

Sample	Replicate	ENA Accession	Number of reads	Number of filtered reads*	Number of mapped reads on MAC**	%	Number of mapped reads on MIC**	%
Input	1	ERS3000371	9430818	7856786	6929340	88.2	7015422	89.3
H3K27me3	1	ERS3000373	6028738	4932700	1465830	29.7	1772716	35.9
H3K4me3	1	ERS3000377	10490550	8039260	4602414	57.2	4466804	55.6
H3K9me3	1	ERS3000375	11608410	8633428	3027432	35.1	5857844	67,9
Input	2	ERS3000372	12244786	10073580	9109940	90.4	9077564	90.1
H3K27me3	2	ERS3000374	9349800	5337322	1549350	29.0	1652260	31.0
H3K4me3	2	ERS3000378	11281436	8840084	5129996	58.0	4979036	56.3
H3K9me3	2	ERS3000376	18933858	14624882	8367024	57.2	12886074	88.1

* Min sequencing quality 20 and no PCR duplicates
** High quality BOWTIE2 mapping (-q 20)

Supplementary Table 5. Description of ChIP-seq data deposited in the European Nucleotide Archive.
The columns provide the sample label, the ENA accession, the total number of reads, the number of filtered reads, the number of filtered reads that were mapped to the MAC reference genome and the number of reads that were mapped to the MIC reference genome assembly. The percentage of mapped reads is with respect to the number of filtered reads in each sample.
