# Supplementary material for: The Polycomb protein Ezl1 mediates H3K9 and H3K27 methylation to repress transposable elements in Paramecium
Source: Nat Commun. 2019 Jun 20;10:2710. doi: 10.1038/s41467-019-10648-5 (PMC6586856; doi:10.1038/s41467-019-10648-5)
Supplement: Supplementary file 3 — Description of Additional Supplementary Files [file 41467_2019_10648_MOESM3_ESM.pdf]

Description of Additional Supplementary Files

File name: Supplementary Data 1

Description: Mass spectrometry determination of differentially modified histone H3 peptides in purified *Paramecium* histone H3 samples obtained from *in vitro* methylation reactions with $Ezl1^{wt}$ and $Ezl1^{H526A}$ proteins.

File name: Supplementary Data 2

Description: List of DE genes upon *EZL1* RNAi.
